# To Treat or Not to Treat? Polish Physicians’ Opinions about the Clinical Aspects of Cannabinoids—An Online Survey

**DOI:** 10.3390/jcm11010236

**Published:** 2022-01-01

**Authors:** Martyna Joanna Hordowicz, Jerzy Jarosz, Anna Klimkiewicz, Małgorzata Czaplińska, Agnieszka Leonhard, Maria Wysocka

**Affiliations:** 1 Hospice of St. Christopher in Warsaw, 02-781 Warsaw, Poland; m.czaplinska.2012@gmail.com (M.C.); aleonhard@op.pl (A.L.); maria.wysocka@fho.org.pl (M.W.); 2 Polish Society of Medical Cannabis and Cannabinoids, 02-781 Warsaw, Poland; jerzy.jarosz@op.pl (J.J.); anna.klimkiewicz@wum.edu.pl (A.K.); 3 Department of Psychiatry, Medical University of Warsaw, 02-091 Warsaw, Poland; 4 Department of Clinical Nursing, Medical University of Warsaw, 02-091 Warsaw, Poland; 5 Department of Medical Ethics and Palliative Medicine, Medical University of Warsaw, Litewska 14a, 00-581 Warsaw, Poland

**Keywords:** physicians, opinion, medical cannabis, cannabinoids, Polish, clinical aspects

## Abstract

Introduction: Medical cannabis’ importance in Poland increased dramatically following its legalization as the 12th country in Europe in 2017. However, no studies have been published to give insight into Polish physicians’ opinions about medical cannabis. Objectives: To investigate physician’s opinions about cannabinoids’ utility in clinical practice, concerns regarding their safety profile, and their clinical experience with cannabinoids. Methods: The survey using a self-developed tool was conducted online; participants were physicians with or without specialist training. Participation was voluntary. Physicians were recruited through personal networks, palliative care courses, and Medical Chambers. Results: From June to October 2020, we recruited 173 physicians from 15/16 voivodeships. The largest age group (43.9%; *n* = 76) was 30–39 year-olds. A similar proportion declared they never used cannabis and did not receive any training regarding cannabinoids (60% for both). Only 15 (8%) ever prescribed medical cannabis, although about 50% declared knowing suitable patients for such therapy, and 53.8% had at least one patient proactively asking for such treatment in the last 6 mo. The most common indication chosen was pain: chronic cancer-related (*n* = 128), chronic non-cancer (*n* = 77), and neuropathic (*n* = 60). Other commonly chosen conditions were alleviation of cancer treatment side-effects (*n* = 56) and cachexia (*n* = 57). The overall safety profile of THC was assessed as similar to most commonly used medications, including opioids; NSAIDs and benzodiazepines were, however, perceived as safer. Conclusions: Polish physicians favored the legalization of medical cannabis. However, it is of concern that a limited number have any experience with prescribing cannabis. The creation of clear guidelines to advise physicians in their routine practice and education about pain management and the risks related to the consumption of recreational cannabis for medical conditions are needed.

## 1. Introduction

The use of cannabinoids by humans for alleviating pain and other symptoms dates back to 5000 y ago [1]. Western medicine has used cannabinoids from the XIX Century until the mid-XX Century when *Cannabis sativa*-based tinctures and extracts spread thanks to Sir William O’Shaughnessy, Sir William Gowers, and other physicians. Its use was propagated in treating various conditions, including epileptic seizures, muscle spasms in the course of tetanus, Parkinson’s disease, and migraines [2].

Cannabis sativa contains over 100 phytocannabinoids, which are most important from the medical and legislative point of view [3]. Cannabis, including the industrial type, bears the stigma of a dangerous drug, a serious hazard to human health [4,5]. International laws introduced throughout the XX Century oblige European countries to introduce control measures over psychoactive substances. The 1961 Convention of the United Nations set principles for *Cannabis sativa* cultivation for purposes other than industrial, whereas the 1971 Convention established standards for control over tetrahydrocannabinol (THC) [5,6]. They created barriers to medical cannabis use in the following decades, although none of these prohibit the use of cannabinoids to treat medical symptoms and conditions per se. However, some organizations have already begun to challenge the status quo. In June 2018, the World Health Organization (WHO) declared in their critical report that that there is no evidence to prove that cannabidiol (CBD) has any abuse potential or causes dependence [7]. Furthermore, the Expert Committee of Drug Dependence (ECDD) of the WHO made several recommendations that might lift some of the restrictions that have halted its medical uses and research. In December 2020, the United Nations (UN) Commission on Narcotic Drugs decided to reclassify *Cannabis sativa,* acknowledging its therapeutic potential [8]. These statements might have created momentum for further changes in policies related to cannabis in countries belonging to the UN, including Poland.

Recently, numerous countries have lifted national restrictions and legalized medical cannabis under diverse legal frameworks [6]. New countries have adapted regulations rapidly, driven by patient organizations, advocacy groups, and medical associations [9]. According to a 2018 survey conducted by the European Pain Federation (EFIC), 21 countries out of 31 countries participating in the survey had made medicines containing cannabinoids available. In contrast, in 2001, the Netherlands was the only such country in Europe [9]. The healthcare community was left unprepared for these sudden changes with no background education and a low availability of reliable, independent sources of knowledge [10]. There is pressure from patients who often have unrealistic expectations regarding treatment with cannabis because they can share general misconceptions about its healing properties.

Although more than three decades have passed since the fall of the Soviet Union and communism in Poland, there are still profound differences in many fields of medicine between countries of the former Soviet bloc and Western Europe. This has been demonstrated by the approach towards other controlled substances, such as opioids [11,12]. For comparison, the average use of opioids measured by defined daily doses for statistical purposes (s-DDD) in Denmark or the Netherlands exceeds 12,000 s-DDD. In Poland, consumption of opioids is lower than 2000 s-DDD, similar to other countries belonging to the former Soviet Union [11].

Poland allowed herbal cannabis for medical purposes in 2017 as a pharmaceutical raw material, and the first strain was approved in late 2018 [13,14]. Sativex, a spray containing tetrahydrocannabinol (THC) and CBD, was registered in 2012 [15]. Before this time, medical cannabis was available solely through targeted import for a single patient [16]. The patient having to pay the full cost of treatment has restricted the number of those who could benefit nationwide. According to 2017 regulations, raw cannabis became a magistral preparation by being dispensed by the pharmacy. It might be used to prepare other formulations, e.g., for topical applications. Just as in the case of all magistral preparations, pharmacists are responsible for dispensing the right drug and instructing the patient about it, as THC is a psychoactive substance [13]. However, there are several problems with this approach. As the Polish Ministry of Health admitted, some critical excipients for preparing magistral preparations are unavailable in Poland. The current Polish (and European) Pharmacopeia has no description of herbal cannabis [17]. Polish pharmacists, therefore, rely solely on the German edition of the Pharmacopeia. The definition of the upper limit of THC that can be dispensed to the patient (quantity sufficient for a maximum of 90 d of treatment) is also vague [14,17]. Additionally, dispensing cannabis in the original package has led to secondary, illicit trading of this on the black market, which contains recreational cannabis sold as the medical one [17]. More importantly, there are no official guidelines created by medical associations or governmental agencies to guide clinicians in implementing cannabinoids into their practice [9].

Given the short timeframe in which cannabinoids in medicine regained popularity worldwide, which has led to their legalization, it becomes urgent to explore the perceptions of the medical community on the clinical aspects of cannabis and cannabinoids. Studies investigating healthcare professionals’ perspectives on cannabinoids give conflicting results. In short, there is no consensus among the medical community, and the opinions about the clinical utility of cannabis are mixed and vary by age, gender, medical specialty, and other factors such as religiousness and the location where the study was conducted. Participants of most of these studies consider the self-assessed level of knowledge insufficient for using cannabinoids in routine clinical practice [18,19]. In some surveys, most physicians claimed that medical cannabis has little or no benefit to human health, while others reported contrary results [19,20,21]. In several studies, the participants voiced concerns about the misuse of cannabis as a recreational drug and opted firmly against decriminalizing cannabis [20,22]. Therefore, collecting insights from different countries and medical professionals with miscellaneous backgrounds, training, and experience seems well justified.

Research involving European healthcare practitioners remains scant, and no studies have been conducted among certified physicians from Central-Eastern Europe. Most of the available literature concerns medical professionals from the Americas—the USA [23,24,25,26,27,28,29], Canada [19,20,30,31,32], and Israel [19]. A systematic review published in 2020 identified only two such surveys conducted among physicians from Europe [18]. The review revealed profound differences between Eastern Europe and Western countries [18]. In surveys conducted among prospective physicians in Belarus and Russia [33,34], they declared much less support for medical applications of cannabinoids and much lower willingness to use them in clinical practice than HCPs from Western countries. By contrast, nursing students from Spain and physicians from Norway declared more support for cannabis legalization for medical purposes [21,35].

A 3 y period following the legalization of medical cannabis in Poland should be sufficient to allow for the opinions of physicians to mature and take shape. To date, only research among students of medical faculties has been conducted [36,37]; however, both were designed as a knowledge test and did not investigate opinions, fears, and the general perception of using cannabinoids in clinical practice. Our study is the first conducted in Poland among physicians. It aimed to investigate their perception of cannabinoids’ utility in clinical practice, perceptions and concerns regarding their safety profile, and their clinical experience with cannabinoids. We also aimed to identify possible factors that contributed to their choices.

## 2. Materials and Methods

The survey was conducted online; participants were physicians with or without specialist training. Participation was voluntary, and no incentives were offered to the physicians. Due to the coronavirus pandemic outbreak in March 2020, we decided to conduct the study using a digital platform. The recruiting took place between June and October 2020. We used Google Forms for data collection. The report of this study was based on the recommendations included in the Checklist for Reporting Results of Internet E-Surveys (CHERRIES) [38].

The protocol for this study was prepared concerning the recommendations of the Declaration of Helsinki. It was approved by the Bioethics Committee of the Medical University of Warsaw (IRB statement from 3 February 2020 Number AKBE/22/2020).

### 2.1. Recruitment of Study Participants

Statistically, an average medical doctor in 2017 in Poland was 52 years old [39]. Knowing that the minimal time required to acquire specialist training is 12+ y, most currently practicing physicians attended university in the 1980s. At that time, Poland was still under Soviet influence, which ended after 1989. This age group is less likely to use social media; therefore, we targeted them through Medical Chambers and online courses. Younger physicians were recruited mainly through a professional newsletter and social media. We employed a wide range of sources allowing the participation of doctors from varying medical backgrounds, different age groups, and geographical localization.

Only a direct link could be used to access the questionnaire. We recruited the participants of the study by sharing a link with:

- Regional Medical Chambers in Poland. There are 16 Chambers, 1 in each voivodeship. It is currently mandatory that each medical doctor be a member of one located in his/her primary workplace. Each has its website and a newsletter sent to every member. We sent invitations for participation to all of them;
- Attendees of the online palliative medicine for physicians course organized in the Hospice of St. Christopher in Warsaw. There were two courses in total, run in June and October 2020;
- “Residents Agreement” Facebook group. This is a closed group, and the administrators verify each new member’s professional background. We obtained their permission to share the link with a description of the study aims and information about the researchers’ organization;
- The owners of a newsletter for young medical doctors sent by an organization that helps physicians prepare for professional and specialization exams;
- Among a personal network of physicians from different medical backgrounds.

In order to ensure the anonymity of participants, we did not collect the data allowing for tracking of participants (such as personal and contact data, IP address). We decided that the whole dataset would be reviewed manually to detect double or accidental entries by non-medical professionals and medical professionals and excluded them from the dataset. Identification of individual users was possible thanks to the open-ended questions included in the questionnaire, such as questions about medical specialty, years of practice, and additional specialties. In the case of identifying a person with the same answers, we could further compare answers related to age group, gender, and location to check if we identified a true double entry.

### 2.2. Survey Questions and Development

The complete study questionnaire consisted of 57 items grouped into 5 different topics. This report focused only on those dedicated to clinical practice, preferences regarding cannabinoids’ use, and the perception of safety compared to other available medicines. The parts dedicated to educational needs and systemic solutions were described elsewhere [40].

We included both open (e.g., where we asked the participants to write out their medical background) and closed questions (containing multiple-choice answers and Likert scales). The questions about clinical aspects were adapted from another questionnaire developed for a study commissioned by the National Bureau for Drug Prevention (NBDP).

The survey was tested before study initiation by medical doctors from the research group. Afterward, they corrected the wording of items or the mechanics of the survey, i.e., type of questions where necessary. We made it mandatory to fill out all the fields before the answers could be submitted to avoid incomplete records.

### 2.3. Statistical Analysis

Statistical analysis was performed using the IBM SPSS Statistics ver. 26. The conventional threshold of *p* = 0.05 was used as the level of statistical significance. An analysis of the basic descriptive statistics was performed. In order to answer the research questions, we used basic descriptive statistics, including normality of distribution tests, and Mann–Whitney tests and multinomial logistic regression.

In the first step of the analysis, the distributions of the quantitative variables were checked. For this purpose, basic descriptive statistics and the Kolmogorov–Smirnov test examining the normality of the distribution were calculated. The next step of the statistical analyses tested differences in attitudes toward cannabinoids according to the sociodemographic variables of the physicians studied. For this purpose, Mann–Whitney tests were performed.

Multinomial logistic regression was performed to verify how sociodemographic variables and other characteristics predicted participants’ choices regarding the first-line treatment of pain. The reference group for the dependent variable was the choice of cannabinoids as the first-choice treatment. Pearson’s χ^2^ test was used for this purpose.

## 3. Results

There were 173 physicians from Poland who completed the survey: 86.7% (*n* = 150) were less than 50 years old; 64.7% (*n* = 112) lived in cities with more than 100,000 inhabitants. Participants came from almost all (15 out of 16) voivodeships (macro-regions). Western (*n* = 66; 38.15%), central (*n* = 59; 34.1%), and eastern macro-regions (*n* = 48; 28.78%) were represented by a similar number of participants. Approximately 1/5 (*n* = 36; 20.8%) declared the private sector as the primary workplace.

We present other characteristics of the study participants in Table 1. The most common medical specialties were internal medicine and general practitioners (GPs), oncology-related, and anesthesiologists. It is essential to highlight that one physician might have multiple specialties (some more than three, e.g., internal medicine, pediatrics, GP); therefore, the number in the “medical specialty” section of Table 1 exceeds the total number of participants.

During the review, we did not find any double entries, which can be defined as entries from a person having a title in the same field(s) of medicine, belonging to the same age group, having the same gender, and coming from the same micro-region of Poland.

### 3.1. Clinical Experiences with Cannabinoids

Most participants (91.3%; *n* = 158) declared having no previous clinical experience with cannabinoids. Only four (2.3%) participants had significant clinical experience with cannabinoids and treated more than ten patients, while only eight (4.6%) admitted prescribing cannabinoids in the past. At the same time, almost half of the participants claimed that some of their patients could benefit from cannabinoid therapy (49,7%; *n* = 86). More than half saw at least one patient who asked about cannabinoid therapy in the last 6 mo (53.8%; *n*= 93). More details about the clinical aspects are presented in Table 2.

### 3.2. Indications for Cannabis Use

Physicians chose indications for cannabis use from a multiple-choice list included in the survey. They could choose an unlimited number of conditions in which they would use cannabinoids and added their suggestions to the list. This included both well-studied indications, such as different kinds of pain or epilepsy, and potential indications that require further research (such as most psychiatric conditions).

Conditions selected by participants were in principle related to oncology and terminal care. Only 20 participants declared having no intention to use cannabinoids in clinical practice. The most commonly listed indications were pain, especially chronic cancer-related pain (*n* = 128). A similar number of participants chose chronic non-cancer pain and neuropathic pain (77 and 60 participants, respectively). These conditions were also chosen by the fifteen clinicians who prescribed cannabinoids in the past. Frequently, participants chose cancer-related cachexia (*n* = 57) and chemotherapy-induced nausea and vomiting (CINV) (*n* = 56). In contrast, other common kinds of pain, such as lower back pain (*n* = 25) and joint pain (*n* = 21), were chosen by less than 15% of clinicians.

A smaller proportion of physicians chose diseases from other medical fields as indications for cannabinoids’ use. Psychiatric conditions, such as insomnia (*n* = 22), depression (*n* = 20), anxiety (*n* = 22), and post-traumatic stress disorder (PTSD) (*n* = 15), were among the rarest conditions picked by physicians. furthermore, neurologic conditions, such as multiple sclerosis (*n* = 51), epilepsy (*n* = 40), and Alzheimer’s disease (*n* = 11), were chosen relatively rarely. More details are shown in Table 3.

### 3.3. Evaluation of the Safety Profile of Cannabinoids

We asked to compare a perceived safety profile of THC to other commonly used drugs classes, including controlled substances. We used a five-point Likert scale, where “1” meant that the safety profile was much worse and “5” much better compared to THC.

On average, THC’s safety profile was thought to be similar to weak opioids (tramadol) and buprenorphine. This option was chosen by 74 (42.8%) and 70 participants (40.5%), respectively. Potent opioids’ safety in relation to THC was ranked as three, similar to THC (*n* = 65; 37.8%), “(much) better than THC” (*n* = 56; 32.4%), and “(much) worse than THC” (*n* = 52; 30.1%) by a similar number of participants.

The only classes of drugs whose safety profile was assessed as better than THC were non-steroid anti-inflammatory drugs (NSAIDs), acetaminophen, and benzodiazepines (BDZs), where the most commonly picked answer was “4—better than THC”. However, the median was “3—similar to THC” in both cases. The full results are presented in Table 4.

To evaluate the clinical significance of THC’s most common side-effects, we asked the participants to pick those clinically meaningful in their opinion, then rank them on a scale from one to five: “1” marked a clinically nonsignificant problem, whereas “5” was reserved for the most significant.

The possibility of triggering psychotic symptoms and worsening psychiatric conditions was chosen by 92% and 91.9% of participants, respectively. However, psychotic disorders were most often claimed as a clinically significant problem (mode = 5; 40.5% of the total number of participants), whereas worsening psychiatric conditions were most often considered “rather significant” (mode = 4; *n* = 25).

Short-lasting adverse effects of THC such as euphoria, motor impairment, vertigo and dizziness, and sedation were chosen by 85.5%, 89%, 84.4%, and 82.7%, respectively. Their impact was most often ranked as “moderate” (mode = 3), except euphoria, which was not thought to be a major problem (mode = 1). Immediate adverse effects of THC were chosen by a smaller proportion of participants than psychiatric complications, possible interactions, and developmental issues, including low body weight (LBW) and brain development. More details can be found in Table 5.

### 3.4. Attitudes towards Cannabis Legalization

We investigated the point of view of medical cannabis legalization and use and factors that might influence doctors’ opinions. We applied a five-point Likert scale from one (strongly disagree) to five (strongly agree).

In total, Polish physicians favored legalizing medical cannabis and declared that they would want to use it for self-treatment or their family members (median for both—5.0, “strongly agree”). Nonetheless, they were less supportive of increasing the number of available medicines containing cannabinoids (median—4.0, “agree”).

We also tested for differences between physicians in subgroups. Groups were identified by gender, age, work location (small vs. larger cities), primary work sector (public vs. private), participation in educational activities, and contact with people with addiction in clinical practice. Mann–Whitney tests were performed for each subgroup.

Personal use of recreational cannabis was a factor that statistically significantly influenced attitudes toward legalization, use, and increasing the diversity of products containing cannabis in Poland (*p* < 0.001 for each comparison). Age also proved to be a differentiating factor in attitudes toward cannabinoids. Comparisons were made between physicians under 50 years of age and physicians 50 years of age and older. Younger physicians expressed more positive attitudes toward cannabinoids, i.e., their use for treating close relatives (*p* = 0.036) and legalization of medical marijuana (*p* = 0.009), than older physicians. Similarly, physicians from larger cities (with a population of at least 100,000) were more likely to use cannabinoids to treat themselves or their family members and more supportive of legalizing marijuana for medical purposes than physicians from smaller cities. These differences were also statistically significant (*p* = 0.046 and *p* = 0.036, respectively). For physicians who have contact with people addicted to drugs or psychoactive substances, compared to physicians without such contact, the results were statistically significant only for the question about the number and variety of these cannabis-based medicinal products should be greater (*p* = 0.042).

No statistically significant differences were found between the genders as a factor differentiating attitudes towards cannabinoids (for all questions, *p* > 0.05) and similarly when comparing physicians working primarily in the public and private sectors and physicians who had attended any lecture or training on the medical uses of cannabinoids (*p* > 0.05 for all comparisons).

All comparisons are presented in Table 6.

### 3.5. First Choice Treatment

We also asked about the first-choice treatment in the case of intense pain for participants and relatives. The majority opted for the selection of opioids (*n* = 98; 56.6%); however, a significant proportion also chose cannabinoids (*n* = 48; 27.7%). Therefore, we decided to investigate whether any factors might have influenced such choices.

Multinomial logistic regression was performed. The reference group for the dependent variable was the choice of cannabinoids as the first-choice treatment. Pearson’s χ^2^ test was used for this purpose. The analysis showed a satisfactory fit according to both tests (Pearson’s chi-squared: χ^2^(106) = 125.20; *p* = 0.098; variance: χ^2^(106) = 125.51; *p* = 0.130). In contrast, the model with all predictors was not significantly different in terms of data fit from the model containing only a constant: χ^2^(14) = 15.75; *p* = 0.329; Nagelkerke R2 = 0.10.

Despite the statistically insignificant model, past cannabinoid use emerged as a significant predictor for choosing cannabinoids vs. opioid medications as the first-line treatment. If study physicians had used recreational cannabinoids in the past, they were 2.17-times more likely to choose cannabinoids over opioid medications as the first-line treatment for themselves and their relatives than if they had no experience with cannabinoids. The results are shown in Table 7.

## 4. Discussion

Medical cannabis’ clinical importance in Poland increased dramatically following its legalization in 2017 as the 12th country in Europe [13]. It is expected that Poland will play a crucial role in expanding the European cannabis market. Even before the legalization of THC-containing cannabis, there had been an increase in interest in industrial hemp-based products. The demand was created through intensive marketing of CBD oils, cosmetics, food, and other products. According to the 2019 report, it is estimated that the total value of the cannabis market will reach USD 2 billion in 2028 [41].

The Polish legal framework in which cannabis is considered a raw pharmaceutical material is unique in Europe [42]. On the one hand, it implies no restrictions for cannabis use in terms of gender, symptoms, or age. However, hospice and hospital pharmacies cannot dispense cannabis because they cannot make magistral preparations, limiting access to an important target group, namely palliative patients [13,17]. Additionally, there are no approved indications list, contraindications, or national recommendations to define the dosing and other vital aspects for making magistral preparations of adequate quality [40].

Given the complex legislative solutions and growing patient demand, increasing the awareness, knowledge, and skills among prescribing physicians seems necessary. Although physicians generally favored cannabis legalization, 91.3% declared they had no (or minimal) clinical experience with cannabinoids. At the same time, 49.7% declared they had patients for whom cannabinoids might be beneficial, and 53.8% of participants admitted they had at least one patient asking about cannabinoid therapy in the last 6 mo. While medical doctors declared they would use cannabinoids for pain treatment (77/173—chronic non-cancer pain; 60/173—neuropathic pain) and cancer-related symptoms (pain—128/173; 57—cachexia related to cancer; CINV and other—56/173), only 15 of them had prescribed cannabis in the past.

Notably, on average, Polish physicians agreed with the statement that the number of products containing medical cannabis should be increased and strongly agreed that the legalization of cannabis is the right move. More than a quarter also declared that cannabinoids would be the first-choice treatment for high-intensity pain for themselves or their relatives. We also confirmed that past cannabis use increased the odds for selecting cannabinoids in these instances and favoring legalization. We found that younger physicians were more open to legalization and increasing the number of products and use of cannabis in the treatment of their relatives. However, an Irish study among general practitioners found the opposite effect of age, and older physicians were more inclined towards medical cannabis [22]. Furthermore, Crowley et al. found that those with training in addictions declared more support for medical uses of cannabis, and we observed a contrary effect [22]. Other reports have suggested that gender might also play a role in perspectives on cannabis, but we did not observe such a correlation [21,22,33]. Surprisingly, doctors working primarily in the private sector were not different from those in the public sector. Knowing that patients pay the total cost of treatment, those who can afford such therapy would rather use private healthcare, which is easier to access and offers a shorter waiting time for a consult than public healthcare. Nonetheless, the sector of work was not found to influence physicians’ perspectives.

It might be concerning that almost 40% of Polish physicians participating in this study had used cannabis in their lifetime. According to the 2019 report by Polish National Office for Counteracting Drug Addiction, 7.8% of the Polish population aged 15 y or more used cannabis in the last year; this makes cannabis the most prevalent illicit drug in Poland [43]. In studies conducted in other countries, the proportion of users was similar. In a 2012 study, the average proportion of the population aged 15 y or more declaring cannabis use in the EU for the adult population was 23.3%, ranging from 1.6% in Romania to 40.9% in France [44]. This is higher than the population’s average for Poland (12.2%) and lower than that found in our study [44]. Nonetheless, in other studies, such discrepancies between the official reports were also identified. In a study by Perreira et al., 48.9% of nursing students from Spain had used cannabis. In contrast, in official statistics, it reaches 30% [35]. In a study conducted among medical students from Serbia, a country that used to be a part of the Soviet Union, the proportion of users of cannabis also exceeded the one found in official reports similarly to our findings (34.9% vs. 12.4%) [45]. A plausible explanation is that most of our participants were from larger cities, where illicit substances are more accessible. In contrast, medicine students from other countries from the former Soviet Union, such as Belarus and Russia, rarely consume cannabis for recreational purposes (10.6% and 3.3%, respectively) [33,34]. That might indicate some social changes arising between counties of the former Soviet bloc: some are now drifting towards the Western countries of Europe, and some are more inclined towards the East. Nonetheless, other possible explanations could be investigated in other research.

Past use of recreational cannabis is an important factor influencing perspectives on medical cannabis in numerous studies. We found that it increases the odds of using cannabinoids as the first-choice therapy for severe pain by 2.17-times. It was also shown that these physicians presented a more positive attitude toward legalization, use in the treatment of their relatives or themselves, and increasing the number of cannabinoid-containing medicines in Poland (*p* < 0.001 for comparison with nonusers). In other studies, it was also demonstrated that this factor has a significant influence on opinions. In a study among nursing students in Spain, users were more inclined towards medical cannabis legalization than nonusers (85.4% vs. 72.6%; *p* = 0.003), as well as recreational (44.3% vs. 17.7%; *p* < 0.0001) [35]. In Belarus, medicine students who used marijuana more often declared they would recommend cannabis to patients in the case it was legal (85.9%; 61.8%; *p* < 0.001) and more rarely admitted that it could be addictive (33.9%; 12.1%; *p* < 0.001), that it has the potential to cause serious side-effects, and is dangerous for physical (53.1%; 27.9%; *p* < 0.001) and mental health (55.6%; 23.3%; *p* < 0.001) [45]. A similar relationship was identified in Serbia for medical and recreational cannabis legalization [34].

In the authors’ opinion, Polish physicians’ optimistic attitude towards the legalization of medical cannabis is a positive finding, given that pain is one of the most common qualifying conditions for such treatment in numerous reports from patient registries [46,47,48]. Pain treatment was also the most frequently chosen indication for using cannabinoids by Polish physicians. In previous studies involving physicians, it was consistently ranked among the best-known indications for cannabis use, together with palliative care and multiple sclerosis [18,19]. Sixty-three percent of Irish GPs agreed that cannabis is a legitimate treatment option for chronic pain, and almost forty percent of Norwegian physicians believe that its addition to the treatment schedule can reduce opioid consumption [21,22]. Moreover, almost 80% of primary care physicians from Minnesota agreed that cannabis should be offered for cancer pain and almost 70% for both pain as a symptom and pain refractory to treatment [24]. A similar proportion was found among physicians from New York [23]. In another study comparing the perceptions of clinicians and patients from San Francisco, both groups admitted that cannabis could alleviate pain, at least to some extent, and both groups were less concerned about co-use of cannabis and prescription pain medication than cocaine or opioid abuse [27]. The clinicians in that study reported that they were less likely to ask or conduct cannabinoid tests than for other controlled substances.

Despite the openness and encouraging attitude towards medical cannabis use, a limited proportion (8%; *n* = 15/173) have ever issued a prescription. Similarly, access to adequate pain treatment in Poland is limited, as evidenced by official reports; however, it is estimated that 20% of the 38 mln Polish population lives with chronic pain [49]. That topic was raised on numerous occasions by the Ombudsman, Patient Ombudsman, and parliamentary interpellations [16,49]. The National Institute of Audit published a report revealing that in 94% of hospitals inspected, no evidence of regular pain evaluation was documented, and almost 70% did not establish any procedures to treat pain effectively, including patients undergoing surgical interventions. In addition, they revealed that the inhabitants of 266 out of 373 counties that were distinguished in Poland in 2016 did not have access to public pain treatment centers [49,50]. While countries of the West report higher use and aim to introduce more control measures in the case of opioids, in Poland, many patients struggle to receive adequate medication for pain of strong intensity [11,12]. The bad reputation of opioids makes them stigmatized by society, including healthcare professionals, which was raised by one of the regional consultants in palliative care in an open letter in 2017 [51].

When access to pain treatment is limited and the knowledge about controlled substances is low among healthcare professionals, this might have severe consequences for patients who decide to self-medicate to alleviate their symptoms. It has been observed that patients devoid of professional pain treatment tend to overuse over-the-counter (OTC) pain relievers, mainly non-steroid anti-inflammatory drugs (NSAIDs) and paracetamol. In a study conducted by the Chief Statistical Office, 33% of surveyed Polish citizens took OTC pain medication in the previous 14 d and a similar proportion OTCs for the common cold and its symptoms [52]. In most cases, these medications also contain acetaminophen and ibuprofen (or other NSAIDs). According to a 2016 paper, ¼ admits to modifying the recommended doses of NSAIDs [53]. The poisonings with OTC pain medication, namely acetaminophen, are responsible for up to 70% of acute liver failure cases in Europe and more than 50% of drug-induced liver damage [54]. This class of drugs was considered safer than THC in our study, although the consequences of an overdose of NSAIDs might be severe and even fatal, while most side-effects for medical users of cannabinoids are short-lasting, and a fatal overdose is extremely unlikely [53,54,55]. The OTC status of some pain medications might give a false sense of safety to both patients and healthcare workers, and the stigma surrounding pain medications based on controlled substances leads to suboptimal prescription rates [11]. This leads to inadequate pain treatment (or self-treatment with OTC medications) for severely ill patients and overdose-related side-effects [11,52,53,54,55]. We believe that the introduction of medical cannabis is a reasonable opportunity to initiate comprehensive education for physicians on pain management, which would also help identify the right place for cannabinoids in daily practice.

An essential complication for a smooth introduction of medical cannabis into regular clinical practice is the lack of consensus of medical society in its recommendations. In the case of Poland, there is a lack of local recommendations that would consider local specifics [9,40]. Although pain treatment is the most studied and least controversial of the indications for cannabis use, its place in clinical practice is yet to be defined. Available recommendations in Europe are primarily heterogeneous, even for indications without mounting evidence to support cannabis use, such as pain [9,56,57,58]. The British National Institute for Health and Care Excellence (NICE) and the Finnish Medical Association have stated that they are not in support of the use of cannabis-based products for pain, whereas EFIC, the Dutch Office of Medicinal Cannabis (OMC), and the German Pain Society list chronic pain as one of the possible indications for use, especially neuropathic and cancer-related pain [9,56,57]. Still, the recommendations from these organizations are different in many aspects, with some restricting cannabis use to unspecified, refractory cases, while EFIC proposes cannabinoids as the third-line treatment in neuropathic pain, after gabapentinoids and lidocaine [58]. Although a considerable population could potentially benefit from using cannabinoids, the availability of such medications remains limited for many patients [59,60]. That could be improved by creating clear recommendations, standardizing the use of cannabinoids, acknowledging local legislative solutions, and educating healthcare professionals involved in patient care.

According to physicians‘ declarations, our results and previous studies might give the illusion that there are few (if any) barriers to cannabinoids’ use for the most studied indications. A lack of experience is one of them. Most participants (91.3%) reported having no clinical experience with cannabis. Furthermore, Arnfinsen et al. demonstrated that only four out of three-hundred thirty-four doctors ever recommended cannabis to a patient [21]. However, data from other papers revealed that there might be other factors than personal opinion and experiences that limit access to medical cannabis [18,19]. We hypothesize that one of them is a lack of awareness by medical professionals of the hazards related to illegal cannabis consumption and the perception that prescribing such a prevalent drug is not a burning need. Patients who are denied access to such treatment turn to illegal sources, creating a gap between the number of official prescriptions and the number of people declaring the use of cannabis for medical reasons. Discrepancies between the number of cannabis users and prescriptions are becoming evident in other countries. In the U.K., 2 y after the legalization of cannabis for medical purposes, only 60 prescriptions in total were issued, whereas the target population was estimated at 1.5 mln (2% of the total population) [59,61]. In The Netherlands, only 16,000 patients benefited from the Medical Cannabis Programme from its beginning until 2016 [62]. At the same time, other reports demonstrated that numerous users of recreational cannabis take it for medical reasons [61]. That is disturbing as recreational cannabis of unknown sources is likely to be contaminated with herbicides, pesticides, heavy metals, and other substances absorbed to the lungs when inhaled [63,64]. In contrast, pharmaceutical-grade cannabis is routinely tested to exclude its presence.

In recent years, a new hazard has emerged of so-called “designer drugs”. Synthetic cannabinoids and other new psychoactive substances (NPSs) are sold as herbal cannabis on the black market. Usually, herbal material is sprayed with these substances [65]. Some of them, such as “Spice” or “K2”, are now widely available in Europe [66]. It is worth highlighting that the patients declare that they would prefer to have qualified physicians’ supervision and obtain a legal prescription; this has been observed in the authors’ clinical practice [61]. Considering that the risk associated with cannabinoids’ use was considered equal to the most commonly used classes of medicines by Polish physicians, education about the potential health hazards related to the consumption of illegal cannabis (including medical purposes) is vital.

Although it is likely a coincidence, in 2017, the same year when the use of medical cannabis was permitted, the Patient Rights Act was updated with a statement that each patient has the right to proper pain management [16]. The Ministry of Health draft legislation debated at that time, which was meant to improve pain management in patients, did not come into force [49]. A new project prepared in 2020 was also meant to standardize the management of patients with pain, but was not welcomed by the medical society because of the extra workload and no additional financing [67,68]. However, our study revealed that Polish physicians are open to new options for managing pain and other symptoms that cannabinoids might alleviate. They also declared that the safety profile is comparable to other commonly used classes of medication. In summary, it becomes urgent to enable Polish patients to access adequate pain treatment by increasing the number of trained medical professionals and medical facilities that would provide medical advice and care.

This study has certain limitations. One of the most important is the small sample size; therefore, we did not recruit enough representatives of different medical specialties to enable subgroup analysis. We did not include nurses, pharmacists, and representatives of other medical professions involved in direct patient care. The correlation between past cannabis use and a more positive attitude towards cannabinoids found in our study might have influenced the overall result. Nonetheless, we demonstrated that the nonusers (60% in our group), when considered separately, were also in favor of medical cannabis legalization and use in the treatment of relatives.

## 5. Conclusions

This was the first study to investigate the perspectives of Polish physicians about medical cannabis in both clinical aspects and their educational needs and opinions about systemic solutions (reported elsewhere) [40]. On average, they favored allowing medical cannabis use, regardless of age, gender, professional background, and past experiences with cannabis, but the magnitude of such support varied slightly among some of these groups. Nevertheless, it is of concern that a limited number has any experience with prescribing cannabis, given that most have patients actively asking for such treatment. Awareness of the safety profile of THC is low. Our results might indicate that there is a need to create tools, such as clinical guidelines, to advise physicians in their routine practice. More research could shed light on the differences among representatives of different medical backgrounds and other medical professions.

## Figures and Tables

**Table 1:** Study participant’s characteristics.

		N	%
Age group	<30 years	41	23.7
	30–39 years	76	43.9
	40–49 years	33	19.1
	50–65 years	23	13.3
Gender	Male	59	34.1
	Female	114	65.9
Medical specialty	Internal medicine (and associated specialties) and general practitioners (GPs)	58	
	Oncology and hematology (e.g., radiotherapy, oncology surgery)	16	
	Anesthesiology and intensive therapy	16	
	Psychiatry	11	
	Neurology (adult and ped)	7	
	Palliative care	3	
	None/during medical training (unspecified) ^b^	28	
	Other ^c^	42	
	Medical internship	5	
Primary workplace	Town/villages up to 10,000 habitants	12	9
	Towns from 10,000–20,000 habitants	15	8.7
	Cities 20,000–50,000 habitats	9	5.2
	Larger cities 50,000–100,000 habitants	25	14.5
	Large cities > 100,000 habitants	112	64.7
Primary sector of work	Public	136	78.6
	Private	36	20.8
	No data	1	0.6
Contact with persons with addictions in clinical practice	Yes	76	43.9
	No	97	56.1
Declare use of recreational cannabis in the past	Users	68	39.9
	Nonusers	105	60.7
Obtained any professional education in medical uses of cannabinoids ^a^	Yes	69	39.9
	No	104	60.1

GP—general practitioner. ^a^ Includes a course about cannabinoids for healthcare workers, participation in a lecture about cannabinoids, and/or a conference for medical professionals dedicated solely to cannabinoids and other forms. ^b^ Includes participants during specialist training, but who did not declare in which medical field (*n* = 20) and those who did not have any specialist title (*n* = 6). Doctors without specialist training are allowed to practice general medicine. This group also includes two dentists (who, according to local regulations, are also medical doctors, but have a different scope of authority). ^c^ Includes a variety of medical specialists; in most cases, these were sole representatives of this medical field. Examples include: “radiotherapy”, “emergency medicine”, nuclear medicine”, and “balneotherapy”.

**Table 2:** Clinical aspects of medical cannabis and cannabinoids.

	Answer	N (%)
In your clinical practice, do you have contact with patients addicted to drugs/psychoactive substances?	No	19 (11%)
	Rather not	57 (39.9%)
	Yes, but not regularly	70 (40.5%)
	Yes, often	27 (15.6%)
How many patients asked about medical cannabis/cannabis oil/nabiximols or other medicines containing cannabinoids in the last six months?	None	80 (46.2%)
	1–5	72 (41.6%)
	5–10	12 (6.9%)
	Ten or more	6 (3.5%)
	Numerous patients (much more than 10)	3 (1.7%)
Do you have clinical experience with cannabinoids?	None/minimal experience	158 (91.3%)
	Yes, I have used cannabis to treat up to 10 cases	11 (6.4%)
	Yes, I have used cannabis to treat more than 10 patients	4 (2.3%)
In your clinical practice, do you prescribe CCMs?	No	152 (87.9%)
	Rather not	9 (5.2%)
	Neither agree nor disagree	4 (2.3%)
	Rather yes	0 (0%)
	Yes	8 (4.6%)
Do you have patients who could benefit from treatment with cannabinoids in your clinical practice?	No	32 (18.5%)
	Rather not	19 (11%)
	Neither agree nor disagree	36 (20.8%)
	Rather yes	42 (24.3%)
	Yes	44 (25.4%)

**Table 3:** Indications for cannabinoids’ use according to survey participants.

Indications for Cannabinoids’ Use	If I Were to Use Cannabinoids in My Clinical Practice in the Future, I Would Use Them in the Following Indications (N = 173) ^a^	I Have Prescribed Cannabinoids in My Clinical Practice in the Past in the Following Conditions (N = 15)
Chronic cancer pain	128	5
Chronic non-cancer pain	77	1
Neuropathic pain	60	2
Cachexia related to cancer	57	1
CINV and other cancer treatment complications	56	2
Multiple sclerosis	51	
Spasticity	44	1
Epilepsy	40	
Fibromyalgia	35	1
Cachexia related to AIDS	26	
Lower back pain	25	1
Anxiety disorders	22	
Insomnia	22	
Parkinson’s disease	22	
Joint pain	21	1
Depression	20	
I am not using/not going to use cannabinoids in my clinical practice ^b^	20	
Tourette’s syndrome	16	
PTSD	15	
inflammatory bowel disease	14	
Alzheimer’s disease	11	
Pelvic pain	1	
Migraine	1	

CINV—chemotherapy-induced nausea and vomiting; PTSD—post-traumatic stress disorder; AIDS—acquired immunodeficiency syndrome. ^a^ Doctors declaring prescribing cannabis in the past could also answer a question about potential future uses; the list about future uses was different (more robust) than on past uses. ^b^ Doctors could write an explanation giving various reasons, e.g., “I work with children”, “I am a radiologist”, or “I do not feel qualified enough”.

**Table 4:** Safety of cannabinoids in comparison with other classes of drugs.

How do You Perceive the Safety of THC in Comparison with…	1—Much Worse than THC	2—Worse than THC	3—Similar to THC	4—Better than THC	5—Much Better than THC	Mean	STD (±)	Median	Mode
antipsychotics	12	34	84	34	9	2.97	0.94	3	3
SSRI	14	37	76	34	12	2.96	1.01	3	3
SNRI	14	35	82	32	10	2.94	0.97	3	3
NSAIDs	13	45	50	54	11	3.03	1.06	3	4
acetaminophen	15	39	48	51	20	3.13	1.15	3	4
tramadol	19	27	74	42	11	2.99	1.05	3	3
buprenorphine	16	34	70	46	7	2.97	1	3	3
Strong opioids (other than buprenorphine)	16	36	65	41	15	3.02	1.08	3	3
TCAs including amitriptyline	15	40	67	42	9	2.94	1.02	3	3
BDZs	24	35	45	49	20	3.03	1.23	3	4
gabapentinoids	10	31	89	37	6	2.99	0.88	3	3
Z-drugs	15	40	56	50	12	3.02	1.07	3	3

THC—tetrahydrocannabinol; NSAIDs—non-steroid anti-inflammatory drugs; TCA—tricyclic antidepressants; BDZs—benzodiazepines; Z-drugs—nonbenzodiazepines.

**Table 5:** Clinical significance (and level of) of potential adverse effects of cannabis.

	Euphoria or High	Motor Impairment	Vertigo and Dizziness	Psychosis and Psychotic Disorders	Sedation	Addiction	Interactions with Other Drugs and Substances	Worsening of Psychiatric Conditions	Negative Impact on Brain Development	LBW (in Case of Use in Pregnancy)
Unchosen	25 (14.5%)	19 (11.0%)	27 (15.6%)	14 (8.0%)	30 (17.3%)	22 (12.7%)	17 (9.8%)	14 (8.1%)	25 (14.5%)	35 (20.2%)
1—the least significant	47 (27.2%)	9 (5.2%)	11 (6.4%)	10 (5.8%)	14 (8.1%)	25 (14.5%)	13 (7.5%)	9 (5.2%)	16 (9.2%)	16 (9.2%)
2—rather not significant	42 (24.3%)	38 (22.0%)	30 (17.3%)	13 (7.5%)	27 (5.6%)	35 (20.2%)	23 (13.3%)	15 (8.7%)	12 (6.9%)	14 (8.1%)
3—moderately significant	33 (19.1%)	41 (23.7%)	52 (30.1%)	30 (17.3%)	51 (29.5%)	34 (19.7%)	43 (24.9%)	32 (18.5%)	42 (24.3%)	37 (21.4%)
4—rather significant	15 (8.7%)	40 (23.1%)	40 (23.1%)	36 (20.8%)	35 (20.2%)	34 (19.7%)	53 (30.6%)	69 (39.9%)	34 (19.7%)	37 (21.4%)
5—the most significant	11 (6.7%)	26 (15%)	13 (7.5%)	70 (40.5%)	16 (9.2%)	23 (14.3%)	24 (13.9%)	34 (19.7%)	44 (25.4%)	34 (19.7%)
Median	2.00	3.00	3.00	4.00	3.00	3.00	3.00	4.00	4.00	4.00
Mean	2.33	3.23	3.10	3.90	3.08	2.97	3.33	3.65	3.53	3.43
SD	1231	1170	1066	1233	1129	1319	1155	1091	1291	1284
Mode	1	3	3	5	3	2	4	4	5	3 ^a^

^a^ There was more than one mode; the lowest value is given in the table. CNS—central nervous system; LBW—low body weight.

**Table 6:** Factors linked with support for cannabis use in clinical practice and its legalization.

Question	Median		*p*-Value
	All participants		
Would you use cannabinoids in the treatment of yourself or your family members?	5.0		
Do you support the legalization of cannabis for medical purposes?	5.0		
Do you think that the number of registered medical cannabis products should be increased in Poland?	4.0		
**Gender**	Female (*n* = 114)	Male (*n* = 59)	
Would you use cannabinoids in the treatment of yourself or your family members?	4.0	5.0	0.245
Do you support the legalization of cannabis for medical purposes?	5.0	5.0	0.126
Do you think that the number of registered medical cannabis products should be increased in Poland?	5.0	5.0	0.788
**Age group**	<50 years (*n* = 150)	≥50 years (*n* = 23)	
Would you use cannabinoids in the treatment of yourself or your family members?	4.0	4.0	0.036
Do you support the legalization of cannabis for medical purposes?	5.0	4.0	0.009
Do you think that the number of registered medical cannabis products should be increased in Poland?	5.0	4.0	0.050
**Main localization of work**	Cities < 100,000 habitants (*n* = 61)	Cities ≥ 100,000 habitants (*n* = 112)	
Would you use cannabinoids in the treatment of yourself or your family members?	4.0	4.5	0.046
Do you support the legalization of cannabis for medical purposes?	5.0	5.0	0.036
Do you think that the number of registered medical cannabis products should be increased in Poland?	4.0	5.0	0.062
**Primary work sector**	Working mainly in the public sector (*n* = 136)	Working mainly in the private sector (*n* = 36)	
Would you use cannabinoids in the treatment of yourself or your family members?	4.0	3.5	0.063
Do you support the legalization of cannabis for medical purposes?	5.0	5.0	0.413
Do you think that the number of registered medical cannabis products should be increased in Poland?	5.0	4.5	0.347
**Past cannabis use**	No past cannabis use (*n* = 105)	Past cannabis use (*n* = 68)	
Would you use cannabinoids in the treatment of yourself or your family members?	4.0	5.0	<0.001
Do you support the legalization of cannabis for medical purposes?	5.0	5.0	<0.001
Do you think that the number of registered medical cannabis products should be increased in Poland?	4.0	5.0	<0.001
**Education**	Participated in any medical training/lecture on cannabinoids (*n* = 104)	Did not participate in medical training/lecture on cannabinoids (*n* = 69)	
Would you use cannabinoids in the treatment of yourself or your family members?	4.0	4.0	0.466
Do you support the legalization of cannabis for medical purposes?	5.0	5.0	0.394
Do you think that the number of registered medical cannabis products should be increased in Poland?	5.0	5.0	0.325
**Contact with patients with addiction in clinical practice**	No contact with patients with addictions in clinical practice (*n* = 76)	Contact with patients with addictions in clinical practice (*n* = 97)	
Would you use cannabinoids in the treatment of yourself or your family members?	4.0	4.0	0.343
Do you support the legalization of cannabis for medical purposes?	5.0	5.0	0.207
Do you think that the number of registered medical cannabis products should be increased in Poland?	4.0	5.0	0.042

**Table 7:** Factors correlated with cannabinoids as the first-choice treatment for significant pain (for participants or their relatives) and the OR of using opioids/no medication in reference to cannabinoids.

		*p*	OR (95% CI)
Primary Choice—All Participants			
	Cannabinoids 48 (27.7%)	Opioid medication 98 (56.6%)	None of them 27 (15.6%)
Choosing cannabis vs.			
opioid medication	Gender	0.209	0.62 (0.30; 1.31)
	Age	0.420	0.60 (0.18; 2.07)
	Place of living	0.846	0.92 (0.41; 2.07)
	Public/private sector	0.307	1.66 (0.63; 4.37)
	Prior education	0.323	1.46 (0.69; 3.11)
	Contact with pts with addiction in clinical practice	0.332	0.70 (0.34; 1.45)
	Past use of cannabis	0.046	0.46 (0.22; 0.99)
Neither cannabinoids nor opioids	Gender	0.207	0.52 (0.19; 1.44)
	Age	0.967	0.97 (0.21; 4.58)
	Place of living	0.078	0.40 (0.14; 1.11)
	Public/private sector	0.856	0.88 (0.22; 3.50)
	Prior education	0.714	0.82 (0.29; 2.34)
	Contact with pts with addiction in clinical practice	0.522	0.73 (0.27; 1.94)
	Past use of cannabis	0.558	0.73 (0.26; 2.08)

The results (OR) are presented using cannabinoids as a reference point.

## Data Availability

Source data may be shared upon reasonable request to the corresponding author.
